# Maximum surgical blood order schedule for flap reconstruction in oral and maxillofacial cancer patients

**DOI:** 10.1186/s12903-022-02357-1

**Published:** 2022-08-01

**Authors:** Yili Zhao, Xueer Li, Yuepeng Wang, Yanhong Chen, Dandan Li, Qiming Jiang, Yan Wang

**Affiliations:** 1 Department of Oral and Maxillofacial Surgery, Sun Yat-Sen Memorial Hospital, Sun Yat-Sen University, 107th Yanjiang Xi Road, Guangzhou, 510120 Guangdong China; 2 First Affiliated Hospital of Guangdong Pharmaceutical University, Guangzhou, China

**Keywords:** Oral and maxillofacial cancer, Maximum surgical blood order schedule, Flap reconstruction

## Abstract

**Background:**

We established a MSBOS for flap reconstruction in oral and maxillofacial cancer patients.

**Method:**

We enrolled 2080 cases of oral and maxillofacial flap reconstruction from January 1, 2010 to December 31, 2021. Patient data were collected, including age, sex, BMI, preoperative Hb levels, ASA grade, T stage, flap type, tumor location, and bone flap. Scoring criteria were established based on a multivariate model of independent risk variables and their odds ratios. Two flap-type groups were divided into low-risk, intermediate-risk and high-risk groups by the scoring criteria, and analyzed using univariate and multivariate logistic regression. Perioperative transfusion analysis identified independent risk factors at various Hb levels. The cumulative percentage of patients requiring perioperative blood transfusion for each surgical procedure was calculated to establish the MSBOS.

**Results:**

(1) Regression analysis showed that BMI, tumor T staging, ASA grade, preoperative Hb level (male: Hb < 130 g/L, female: Hb < 120 g/L), and bone flap were independent risk factors for perioperative blood transfusion. (2) Regression analysis showed that independent risk factors for perioperative transfusion included the following: BMI, tumor T3–T4 stage, ASA III, IV grade, and free flap/pediculated flap/bone flap in patients with different Hb levels; T3–T4 stage, ASA grade III–IV in mildly anemic patients; and ASA grade III–IV in moderately anemic patients. (3) A MSBOS was established for flap reconstruction in head and neck cancer patients.

**Conclusion:**

A MSBOS for head and neck cancer procedures was reduced by approximately 30% perioperative blood preparation while ensuring that clinical blood use standards were met. It help optimize blood inventory, and save blood resources.

## Background

Radical tumor resection combined with flap reconstruction is the most effective treatment option for most oral and maxillofacial malignancy patients. However, radical tumor resection and flap reconstruction are always considered high-risk procedures. This is partly due to the particularly high risk of massive blood loss because of the complex anatomic structures and the extensive vascular distribution in the surgical area [1]. Therefore, we need to administer appropriate blood transfusions to ensure patient safety. Hemoglobin (Hb) levels are used to determine the severity of blood loss and whether a transfusion is required. However, no clear standard exists for preoperative blood preparation. Blood transfusion is generally indicated when the Hb level is < 70 g/dL, or there is a 30–40% blood volume loss [1]. As early as the late 1970s, Friedman, an American scholar, first proposed the Maximum Surgical Blood Order Schedule (MSBOS) [2], which aims to optimize the number of units of blood used for each location of primary tumors, improve patients’ safety and reduce treatment costs. Subsequently, as research progressed, countries such as the United States, Japan, India and Australia developed MSBOS for various specialties and achieved good results in patient blood management (PBM) [3–5]. However, these studies mainly focused on orthopedics, general surgery, neurosurgery and other departments [6]. There are few studies on the blood management of malignant tumors, especially head and neck malignant neoplasms.

Blood shortages are a common problem faced by major healthcare systems worldwide, and according to the references reported, inappropriate amounts of blood transfusions have been observed in 4–67% of patients undergoing surgery [3]. An estimated 108 million blood donations are collected worldwide, and more than half of these are collected in high-income countries, home to just 18% of the world’s population [7]. Our study is based on this existing problem. We established a preoperative blood preparation schedule to provide a reliable blood preparation plan for patients with maxillofacial malignancies requiring flap reconstruction, as well as to provide a clinical reference for rational blood allocation and maximize the use of the limited blood resources available. The MSBOS is a table of elective surgical procedures that lists the number of units of blood routinely cross-matched for each procedure preoperatively. We chose to collect and analyze data from 2080 patients who underwent surgery for oral and maxillofacial malignancies in the Department of Oral and Maxillofacial Surgery at Sun Yat Sen Memorial Hospital, Sun Yat-Sen University, from January 1, 2010 to December 31, 2021, to establish a MSBOS for flap repair of oral and maxillofacial malignancies.

## Study design and methods

### Data source and patients

After institutional ethics approval was obtained, data were obtained from 2080 patients requiring flap reconstruction with oral and maxillofacial malignancies from January 1, 2010 to December 31, 2021, at Sun Yet-Sen Memorial Hospital, and the same team of anesthesiologists performed all anesthesia assessments and procedures. To maintain the homogeneity of the study population, patients were excluded from the study if adequate clinical tumor staging or perioperative transfusion data were unavailable. The selection criteria for patients included in the study were as follows: ① age 18–85 years; ② elective surgery; ③ malignant tumors in the jaw, buccal mucosa, oropharynx, floor of the mouth, tongue and gingival areas; ④ flap reconstruction; ⑤ no contraindications to surgery, such as serious cardiopulmonary disorders, hematological disorders and abnormal preoperative coagulation function; and ⑥ no combination of other malignant tumors at the same time.

### Study variables and data collection

Data were obtained from the Sun Yet-Sen Memorial Hospital of Stomatology Patient Information System and the hospital blood bank database. To determine the factors associated with perioperative transfusion in patients with flap reconstruction with oral and maxillofacial malignancies undergoing radical tumor resection, the following factors were compared between the transfusion and non-transfusion groups: patient's medical record number, name, sex, height, weight, diagnosis, type of surgery, American Society of Anesthesiologists (ASA) classification, T-stage in the TNM staging system for malignant tumors (referred to as tumor T stage), preoperative Hb volume (g/L), the volume of blood preparation requested, the actual volume of blood transfused (including intraoperative and 24-h postoperative transfusion of red blood cells), whether the flap graft included a bone flap during surgery, and whether the flap type was a free or pediculated flap. Continuous variables were converted to binary variables using the median as the cutoff point. Clinicopathological characteristics and routine preoperative blood parameters were collected and evaluated to determine their relationship to blood transfusions. The following variables were assessed: age (> 60 years vs. ≤ 60 years), BMI (≥ 18.5 vs. < 18.5), sex, clinical primary tumor extension (clinical T; T3–T4 vs. T1–T2), location of the primary tumor (malignant maxillofacial tumor, buccal mucosa, oropharyngeal, floor of mouth, tongue, gingival sites), ASA score (I, II or III, IV), preoperative Hb level (male: ≤ 130 g/L; female: ≤ 120 g/L), flap type (free flap vs. pediculated flap), and bone flap (osseous vs. nonosseus) (Table 1).

**Table 1:** Association of factors with allogeneic blood transfusion for patients having free-flap surgery

Characteristic	Patients, No. (%)
	(N = 2080)
*Gender*	
Male	1390 (66.8)
Female	690 (33.2)
*Age (years)*	
≤ 60	1309 (62.9)
> 60	771 (37.1)
*BMI*	
Underweight (< 18.5)	1757 (84.5)
Normal and overweight (≥ 18.5)	323 (15.5)
*Preoperative hemoglobin level*	
Normal	1401 (67.4)
Mild anemia	615 (29.6)
Moderate anemia	64 (3.1)
*T stage*	
1	186 (8.9)
2	679 (32.6)
3	364 (17.5)
4	851 (40.9)
*ASA*	
1	55 (2.6)
2	1309 (62.9)
3	661 (31.8)
4	55 (2.6)
*Tumor localization*	
Maxillofacial malignant tumor	305 (14.7)
Buccal mucosa	237 (11.4)
Floor of mouth	220 (10.6)
Oropharynx	154 (7.4)
Tongue	921 (44.3)
Salivary adenocarcinoma	243 (11.7)
*Category of flap*	
Free flap	1104 (53.1)
Pediculated flap	976 (46.9)
*Type of flap reconstruction*	
Osseous	1707 (82.1)
Nonosseous	373 (17.9)

### Statistical analyses

All statistical analyses were performed using the commercially available software, Statistical Package for Social Science (SPSS) version 26.0. The statistical significance level was set at *P* < 0.05. Univariate analysis was performed using the x^2^ test, with a significance level of *P* < 0.05. Univariate analysis using binary logistic regression uncovered independent risk factors for blood transfusion (*p* < 0.05). Multivariate analysis was conducted using logistic regression techniques, with significance determined by odds ratios (ORs) with corresponding 95% confidence intervals (CIs) to evaluate the correlations’ strength. All regression models were constructed using a forced entry method. Continuous variables were dichotomized based on the literature review and clinical judgment.

A scoring criterion was established according to the regression coefficient of independent risk factors. To summarize the scores of different tumor location groups in 2080 patients, the patients were divided into very low-risk, low-risk, moderate-risk, and high-risk groups according to each patient's score.

The same methodology was used for MSBOS establishment as in other clinical disciplines. MSBOS was defined as meeting 90% of the intraoperative patient’s red blood cell transfusion volume [2]. The lowest volume of blood transfused at a cumulative percentage of the actual intraoperative transfusion frequency of ≥ 90% of the enrolled data was used as the recommended maximum preoperative blood preparation for the group.

## Results

In this study, a total of 2080 patients with oral and maxillofacial malignancies who met the inclusion criteria were involved. The demographics and clinical characteristics of patients who underwent radical tumor resection and flap reconstruction are shown in Table 1.

### Univariate analysis of blood transfusion in patients with oral and maxillofacial malignancies

Univariate analysis was used to identify 8 variables significantly associated with an increased risk of exposure to perioperative transfusion. Age (*P* = 0.001), BMI (*P* = 0.000), T stage (*P* = 0.000), ASA grade (*P* = 0.000), tumor location (*P* = 0.015), type of flap (*P* = 0.028), and bone flap (*P* = 0.002) were significantly associated with perioperative transfusions. Results of the Univariable Analysis of Preoperative Factors for Potential Association with Blood Transfusion in Table 2.

**Table 2:** Results of the Univariable Analysis of Preoperative Factors for Potential Association with Blood Transfusion

Variable	Underwent blood	*P* value
	Transfusion	
	No. (%)	
*Gender*		0.832
Male	485 (34.9)	
Female	244 (35.4)	
*Age (years)*		0.001
≤ 60	425 (32.5)	
> 60	304 (39.4)	
*BMI*		0.000
Underweight (< 18.5)	159 (49.2)	
Normal and overweight (≥ 18.5)	570 (32.4)	
*Preoperative hemoglobin level*		0.000
Normal	343 (24.5)	
Mild anemia	331 (53.8)	
Moderate anemia	48 (75.0)	
*T stage*		0.000
1	60 (32.3)	
2	139 (20.5)	
3	91 (25.0)	
4	439 (51.6)	
*ASA*		0.000
1	9 (27.3)	
2	370 (28.0)	
3	331 (47.8)	
4	19 (61.3)	
*Tumor localization*		0.015
Maxillofacial malignant tumor	132 (43.3)	
Buccal mucosa	87 (36.7)	
Floor of mouth	72 (32.7)	
Oropharynx	66 (42.9)	
Tongue	261 (28.3)	
Salivary adenocarcinoma	111 (45.7)	
*Category of flap*		0.028
Free flap	363 (32.9)	
Pediculated flap	366 (37.5)	
*Type of free-flap reconstruction*		0.002
Osseous	157 (42.1)	
Nonosseous	572 (33.5)	

### Multivariate stepwise logistic regression analysis of blood transfusion patients with oral and maxillofacial malignancies

The multivariate stepwise logistic regression analysis of independent risk factors for perioperative blood transfusion are shown in Table 3. The multivariate stepwise logistic regression analysis showed that a BMI of < 18.5, clinical primary tumor extension (clinical T; T3–T4), ASA score (III or IV), low preoperative Hb level, and bone flap were independently associated with an increased risk of perioperative blood transfusion.

**Table 3:** Results of the multivariable stepwise logistic regression analysis

Variable	Regression coefficient	OR (95% CI)	P Value
BMI	0.414	1.513 (1.161–1.972)	0.002
T stage	0.462	1.587 (1.436–1.753)	0.000
ASA	0.65	1.916 (1.587–2.312)	0.000
Preoperative hemoglobin level	1.222	3.395 (2.765–4.170)	0.000
Osseous free-flap reconstruction	0.361	1.434 (1.091–1.887)	0.010

### Multivariate stepwise logistic regression analysis for potential association with blood transfusion in anemia patients

The results of the Multivariate Stepwise Logistic Regression Analysis for Potential Association with Blood Transfusion in Low preoperative Hb patients are shown in Table 4.

**Table 4:** Results of the Univariable Analysis of Preoperative Factors for Potential Association with Blood Transfusion in anemia patients

Variable	Normal preoperative hemoglobin level			Mild anemia			Moderate anemia		
	RC	OR (95% CI)	*P* value	RC	OR (95% CI)	*P* value	RC	OR (95% CI)	*P* value
BMI < 18.5	0.53	1.699 (1.200 2.405)	< 0.05	− 0.14	0.868 (0.628 1.202)	0.394	− 0.67	0.513 (0.143 1.842)	0.306
T3 orT4 stage	0.45	1.572 (1.381 1.790)	< 0.05	0.36	1.434 (1.224 1.680)	< 0.05	0.22	1.245 (0.737 2.103)	0.413
ASA III, IV grade	0.52	1.677 (1.310 2.147)	< 0.05	0.49	1.625 (1.243 2.124)	< 0.05	1.52	4.557 (1.316 15.775)	< 0.05
Tumor localization	− 0.04	0.964 (0.890 1.043)	0.361	− 0.11	0.896 (0.817 0.982)	< 0.05	− 0.11	0.896 (0.646 1.244)	0.514
Free-flap	0.42	1.515 (1.053 2.180)	< 0.05	0.09	1.092 (0.708 1.683)	0.690	0.75	0.045 (0.005 0.373)	< 0.05
Osseous-flap	0.43	1.539 (1.172 2.022)	< 0.05	− 0.48	0.622 (0.451 0.858)	< 0.05	− 3.10	2.108 (0.415 10.704)	0.368

We identified low preoperative Hb levels as an independent risk factor for perioperative transfusion, so we performed a stratified analysis of Hb levels. We further divided patients into three groups according to their Hb levels, including the normal Hb value group (Hb levels: ≤ 130 g/L for males and ≤ 120 g/L for females).), mild anemia group (Hb levels: > 90 g/L, ≤ 130 g/L for males and ≤ 120 g/L for females), and moderate anemia group (Hb levels: between 60 g/L and 90 g/L), where an OR of < 1 suggests that the factor is a protective factor. The multivariate stepwise logistic regression analysis of normal Hb levels showed that a BMI of < 18.5, T stage (T3 or T4), ASA III, IV grade free flap, and bone flap were independently associated with an increased risk of perioperative transfusion. The multivariate stepwise logistic regression analysis of the mild anemia group determined that T stage (T3 or T4) and ASA III and IV grade were independently associated with an increased risk of perioperative transfusion. The moderate anemia group's multivariate stepwise logistic regression analysis showed that ASA III and IV grades were independently associated with an increased risk of perioperative transfusion.

### Establishing a scoring criterion

The regression coefficient is a variable in regression analysis that indicates the magnitude of the effect of the independent variable x on the dependent variable y. A larger regression coefficient indicates a greater effect of x on y. The regression coefficients of preoperative low Hb level (RC = 1.222) were higher than a BMI of < 18.5 (RC = 0.414), T3 or T4 stage (RC = 0.462), ASA IV, III grade (RC = 0.650), bone flap (RC = 0.361), indicating a greater predictive contribution to transfusion risk, therefore a score of 2 for preoperative low Hb level, compared with 1 for the other factors (Table 5).

**Table 5:** Establish scoring criteria according to different risk factors

Independent risk factors	Scoring criteria
Underweight BMI (< 18.5)	1
T stage (T3 OR T4)	1
ASA grade	1
Low preoperative hemoglobin level (Male ≤ 130 g/L; Female ≤ 120 g/L)	2
Osseous flap reconstruction	1

### Transfusion rate for the group of scoring criteria in the group of different tumor locations

Each patient selected for preoperative blood preparation was scored. Each patient's score was grouped for transfusion risk: very low-risk group (0), low-risk group (1–2), moderate-risk group (3–4) and high-risk group (5–6), with higher scores indicating a higher risk of perioperative transfusion for the patients. We collected 2080 patients’ actual blood consumption to determine the transfusion rate for scoring criteria in groups of different tumor locations (Table 6). The data results suggest that the higher the score is for patients with different primary tumor sites, the higher their perioperative transfusion rate, so we can assume that the scoring criteria have some guiding significance. Our analysis yielded perioperative transfusion rates of 26.7%, 30.9%, 58.0% and 71.4% for the very low-risk, status, moderate-risk, and high-risk groups with primary tumor sites of maxillofacial malignancy, respectively. The perioperative transfusion rates for the very low-risk group, low-risk group, moderate-risk group, and high-risk group with buccal cancer were 12.5%, 29.0%, 60.3% and 73.3%, respectively. The perioperative transfusion rates for the very low-risk group, low-risk group, moderate-risk group, and high-risk group for the floor of mouth cancer were 12.0%, 21.7%, 44.3% and 100%, respectively. The perioperative transfusion rates for the very low-risk group, low-risk group, moderate-risk group, and high-risk group with oropharyngeal carcinoma as the primary focus were 26.3%, 30.9%, 63.0% and 87.5%, respectively. The perioperative transfusion rates for the very low-risk group, low-risk group, moderate-risk group, and high-risk group with tongue carcinoma were 12.5%, 29.0%, 60.3% and 73.3%, respectively. The perioperative transfusion rates for the very low-risk, low-risk, moderate-risk, and high-risk groups were 7.9%, 26.7%, 51.4% and 70.0%, respectively. The perioperative transfusion rates for the very low-risk, low-risk, moderate-risk, and high-risk groups for salivary adenocarcinoma were 23.5%, 31.6%, 60.5% and 90.0%, respectively. Among all 2080 patients in this study, the transfusion rate of the very low-risk group was 11.3%, the transfusion rate of the low-risk group was 27.9%, and the transfusion rate of the moderate-risk group was 60.5% and 80.2% in the high-risk group.

**Table 6:** Transfusion rate for the Group of scoring criteria in different tumor localization

	Scoring grouping			
	Very low risk (0) (%)	Low risk (1–2) (%)	Moderate risk (3–4) (%)	High risk (5–6) (%)
Maxillofacial	26.7	30.9	58.0	71.4
Buccal mucosa	12.5	29.0	60.3	73.3
Floor of mouth	12.0	21.7	44.6	100.0
Oropharynx	26.3	30.9	63.0	87.5
Tongue	7.9	26.7	51.4	70.0
Salivary adenocarcinoma	23.5	31.6	93.5	90.0
total tranlation rate	11.3	27.9	60.5	80.2

### Recommended MSBOS in oral and maxillofacial cancer patients with different tumor locations

We divided patients with different tumor primary sites into very low-risk, low-risk, intermediate-risk and high-risk groups based on the score’s total score and the cumulative percentage of transfusion frequency based on the actual amount of blood used in each group. The MSBOS is a table of elective surgical procedures that lists the number of units of blood routinely cross-matched for them preoperatively [8]. The cumulative percentage of the frequency of intraoperative blood transfusion was counted for different groups of patients. When the cumulative percentage exceeded 90% of the minimum blood consumption [5] (i.e., the blood consumption could meet the intraoperative red blood cell transfusion volume of 90% of patients), the transfusion volume was used as the recommended MSBOS for that group. The recommended blood preparation volume for malignant tumors with primary foci located in the maxillofacial region in the very low-risk, low-risk, intermediate-risk, and high-risk groups were 3, 3, 5 and 6 respectively; the recommended blood preparation volume for malignant tumors with primary foci located in the buccal region in the very low-risk, low-risk, intermediate-risk, and high-risk groups were 3, 3, 6 and 5–6, respectively; the recommended blood preparation volume for malignant tumors with primary foci located in the floor of the mouth in the very low-risk, low-risk, intermediate-risk, and high-risk groups were 2–3, 2, 5 and 6, respectively; the recommended blood preparation volume for malignant tumors with primary foci located in the oropharynx in the very low-risk, low-risk, intermediate-risk, and high-risk The recommended blood preparation for malignant tumors with primary foci located in the oropharynx were 2–3, 2, 5 and 6, respectively; the recommended blood preparation for malignant tumors with primary foci located in the oropharynx in the very low-risk group, low-risk group, intermediate-risk group, and high-risk group were 3, 4, 5 and 5–6, respectively; the recommended blood preparation for malignant tumors with primary foci located in the tongue in the very low-risk group, low-risk group, intermediate-risk group, and high-risk group were 2, 4, 4 and 3, respectively; the recommended blood preparation for malignant tumors with primary foci located in the gingiva were 2, 4, 4 and 3, respectively. The recommended blood preparation volume for malignant tumors in the very low-risk group, low-risk group, intermediate-risk group, and the high-risk group were 3, 3, 4 and 8, respectively. The MSBOS of our study covered 90% of the patients in each group (Table 7).

**Table 7:** Recommended MSBOS and cumulative frequency of red blood cell transfusion in Oral and maxillofacial cancer patients with different Tumor localization

	MSBOS recommendation
*Maxillofacial*	
Very low	3
Low	3
Moderate	5
High	6
*Buccal mucosa*	
Very low	3
Low	3
Moderate	6
High	5–6
*Floor of mouth*	
Very low	2–3
Low	2
Moderate	5
High	6
*Oropharynx*	
Very low	3
Low	4
Moderate	5
High	5–6
*Tongue*	
Very low	2
Low	4
Moderate	4
High	3
*Salivary adenocarcinoma*	
Very low	3
Low	3
Moderate	4
High	8

### Actual blood consumption requested blood preparation, recommended blood preparation, original CT value and new CT value of patients with different tumor primary sites

A total of 9083 units were cross-matched for 2080 cases. A total of 2291.5 units of blood were transfused. Our statistical results decrease the new C/T ratio from the original mean of 3.9–3.4. Especially in the very low-risk group for the floor of mouth cancer and the very low-risk group for tongue cancer, the C/T ratio decreased from 11–12 to 5–6. It is worth mentioning that in the high-risk group for maxillofacial malignancies, the C/T value changed from 2.06 to 2.20. The C/T ratio in the high-risk group of buccal cancer changed from 1.77 to 2.04; in the medium-risk group of fundic cancer, the C/T value changed from 2.64 to 3.03 and in the high-risk group from 1.62 to 1.97; in the medium-risk group of oropharyngeal cancer, the C/T ratio changed from 2.38 to 2.49; and in the high-risk group of gingival cancer, the C/T ratio changed from 1.52 to 2.16. In this study, a 30% reduction in preoperative blood preparation was achieved after optimizing the preoperative blood preparation protocol. The C/T ratio for tumor localization according to MSBOS in Table 8.

**Table 8:** The C/T ratio for tumor localization according to MSBOS

Tumor localization	T (%)	Old C/T ratio	New C/T ratio
*Maxillofacial*			
Very low risk	26.7	4.61	3.26
Low risk	30.9	5.15	3.48
Moderate risk	58.0	2.55	2.46
High risk	71.4	2.06	2.20
*Buccal mucosa*			
Very low risk	12.5	6.54	5.29
Low risk	29.0	5.35	3.94
Moderate risk	60.3	1.87	2.55
High risk	73.3	1.77	2.04
*Floor of mouth*			
Very low risk	12.0	11.91	6.96
Low risk	21.7	6.27	3.00
Moderate risk	44.6	2.64	3.03
High risk	100.0	1.62	1.97
*Oropharynx*			
Very low risk	26.3	3.59	2.56
Low risk	30.9	4.48	4.21
Moderate risk	63.0	2.38	2.49
High risk	87.5	1.58	1.38
*Tongue*			
Very low risk	7.9	11.93	5.86
Low risk	26.7	5.11	4.94
Moderate risk	51.4	3.02	2.64
High risk	70.0	2.08	1.18
*Salivary adenocarcinoma*			
Very low risk	23.5	3.47	2.63
Low risk	31.6	5.22	3.88
Moderate risk	93.5	2.72	2.45
High risk	90.0	1.52	2.16
Total C/T ratio		3.91	3.10

## Discussion

For surgeons, preoperative blood preparation and perioperative transfusion are important measures to ensure the successful performance of surgery. Due to the lack of a reference for the amount of blood to be prepared for various procedures, surgeons often request a much larger amount of blood (to be prepared for safety reasons) than the amount expected to be transfused during the operation. Large quantities of cross-matched blood are ordered for surgical patients but rarely end up being used, creating a shortage of reserves and wasting valuable technical time and expensive reagents. Blood banks are faced with an ever-increasing demand for blood and its components, and when this demand exceeds the resources of the blood bank, the surgical plan is compromised. Blood banks need to adopt a blood conservation policy.

Many studies have shown that the introduction of MSBOS has led to significant monetary savings [9]. In the USA, Lowery et al. [10] reported a 70% reduction in blood preparation/transfusion at their hospital after implementing MSBOS, saving approximately 110,000 USD per year. Another hospital in the USA, since the establishment of MSBOS, has reduced blood preparation for elective surgery by 712 crossmatches (33%) after 10 months [11]. In Japan, Yasuda et al. [12] reported a reduction in the blood preparation to transfusion ratio from 5 to 1.5 after the trial establishment of MSBOS. In a large academic medical center, introducing a new preoperative blood preparation catalog reduced blood preparation/transfusion from 2.11 to 1.54 units per procedure in 63,916 surgical procedures in 34 months. In patients who did not require blood preparation (n = 33,216), preoperative blood preparation was reduced by 38% and $137,223 in annual costs for surgical patients [13].

In our study, we found that among the 2080 patients included in the study, we investigated the independent risk factors for perioperative transfusion, which were a BMI of < 18.5 (*P* < 0.002), T3 or T4 classification (*P* < 0.000), ASA III or IV score (*P* < 0.000), low preoperative Hb level (*P* < 0.000), and bone flap (*P* < 0.010) (Table 3). The results of this study were similar to those of Shah [14]. The reported prevalence of perioperative anemia in patients undergoing surgery for cancer ranges from approximately 25–75% [15]. Therefore, we stratified Hb levels into three groups, normal levels, mild anemia, and moderate anemia. We then stratified the independent risk factors for perioperative transfusion in patients with different Hb levels. We found that in the group with normal Hb levels, a BMI of < 18.5 (*P* < 0.05), T3 or T4 stage (*P* < 0.05), ASA grade (*P* < 0.05), free flap (*P* < 0.05), and bone flap (*P* < 0.05) were independent risk factors for perioperative transfusion; in the mild anemia group with Hb levels > 90 g/L, our analysis suggested that T3 or T4 (*P* < 0.05), ASA III or IV (*P* < 0.05) were independent risk factors for perioperative transfusion. In the moderately anemic group with Hb levels of 60–90 g/L, ASA III or IV (*P* < 0.05) was an independent risk factor for perioperative transfusion (Table 4). This result could suggest that when we stratified the analysis by different levels of Hb, excluding the effect of four independent risk factors, namely, BMI level, T stage, ASA score, and bone flap, we found that free flap was also an independent risk factor for perioperative transfusion in patients with head and neck malignancies with normal Hb levels. One reason for this result is, based on the clinician's experience, that this result could be related to the need for microscopic vascular anastomosis of the free flap, a long procedure with a higher risk of bleeding. Another factor could be that as the degree of anemia increases, the need for transfusion increases with lower Hb levels than other factors, resulting in a relative decrease in the impact of other factors.

There are still some shortcomings in our study. When collating the cumulative frequency of actual blood consumption in patients grouped by primary tumor location, there were missing units of blood consumption in some groups, and this part of the data was not collected completely in the clinic. This situation led to the cumulative frequency of patients in the high-risk group with a primary focus on buccal mucosa cancer could not be counted because the number of patients with 5 units of blood was 0. In contrast, the cumulative frequency reached 100% with 6 units of blood, suggesting that a blood preparation of 6 units could meet the surgical blood needs of almost all patients in the high-risk group with buccal cancer, thus leading to our recommendation for blood preparation in increments of 5–6 units. Similarly, in the oropharyngeal cancer high-risk group, the cumulative frequency was not counted because the number of patients with 4 or 5 units of blood was 0. In comparison, the cumulative frequency reached 100% with 6 units of blood, suggesting that 6 units of blood preparation can meet the surgical blood needs of almost all patients in the high-risk oropharyngeal cancer group, thus leading to us recommending blood preparation in increments of 5 to 6 units.

To validate our preoperative blood preparation recommendations, we further counted the original C/T values and new C/T values in patients with different tumor primary sites to correlate as closely as possible the volume of blood cross-matched (C) with the volume of blood transfused (T). The C/T change ratio can be used to monitor the efficiency of the protocol. Some authors suggest that an acceptable C/T ratio should be in the range of 2–3 [8]. The C/T ratio was elevated in some high-risk groups, which may have been caused by insufficient perioperative requests for blood preparation, suggesting a deficiency in current clinical blood allocation.

The MSBOS has been established in many disciplines by matching blood usage according to the surgical approach [10, 11]. After stratifying the Hb levels of 2080 patients in our study, we found that free flap was also an independent risk factor for perioperative transfusion in patients with moderate anemia, which is rarely reported in the literature in some head and neck malignancy surgeries. We also found that among the 4 subgroups derived from the scoring system, the very low We also found that the blood preparation in the very low-risk group, as well as the low-risk group, needed much room for adjustment, thus improving the problem of inadequate blood preparation in the high-risk group at the moderate-risk group level. The MSBOS we established can reduce unnecessary blood preparation and serve as a reference to prevent inadequate blood preparation. Our study focused on the discussion of preoperative blood volume in patients with maxillofacial malignant tumor. In western countries, trauma is the first leading cause of death before the 4th decade of life, as well as the third major cause in patients over 40 years old, being preceded only by cardiovascular diseases and neoplasms [16]. Therefore, the preoperative blood preparation of patients with maxillofacial injury requiring emergency surgery needs to be further studied and it is also the direction of our further research.

## Conclusion

In the 2080 surgical patients we studied, blood preparation was reduced by approximately 30% while ensuring that clinical blood use standards were met. Additionally, our study found that the amount requested was much greater than the amount of blood used in the very low-risk group. In contrast, the amount requested in the moderate- and high-risk groups was often insufficient. In conclusion, our findings can provide a more reasonable plan for clinical blood allocation.

## Data Availability

The datasets generated and analyzed during the current study are not publicly available due to (ownership of data) but are available from the corresponding author on reasonable request.
